# An Immunoinformatics-Based Multi-Peptide Vaccine Provides Antibody-Mediated Protection Against *Acinetobacter baumannii* Infection

**DOI:** 10.3390/vaccines13030236

**Published:** 2025-02-25

**Authors:** Sean Jeffreys, Jadelynn Aki, Megan P. Tompkins, Nicolas D. Prather, Ashlesh K. Murthy, James P. Chambers, M. Neal Guentzel, Chiung-Yu Hung, Bernard P. Arulanandam, Jieh-Juen Yu

**Affiliations:** 1 Department of Molecular Microbiology and Immunology, University of Texas at San Antonio, San Antonio, TX 78249, USA; sean.jeffreys@swid-id.com (S.J.); jadelynnlkaki@utexas.edu (J.A.); meganpt@umich.edu (M.P.T.); nicolas.prather@utsa.edu (N.D.P.); james.chambers@utsa.edu (J.P.C.); m.guentzel@utsa.edu (M.N.G.); chiungyu.hung@utsa.edu (C.-Y.H.); 2 Pfizer, Pearl River, NY 10965, USA; ashlesh.murthy@pfizer.com; 3 Department of Immunology, Tufts University School of Medicine, Boston, MA 02111, USA

**Keywords:** *Acinetobacter baumannii*, antibody, complement, epitope vaccine, Fc receptor, opsonophagocytic killing

## Abstract

Background/Objectives: *Acinetobacter baumannii* is an opportunistic nosocomial pathogen characterized by its multidrug-resistant (MDR) phenotype, increasing patient mortality and healthcare costs as a result. Previously, we constructed an immunoinformatics-based *Acinetobacter* Multi-Epitope Vaccine (AMEV2) candidate and demonstrated robust protection against this MDR pathogen. In this study, we delineate the mechanisms of AMEV2-mediated protective immunity. Methods: In vivo passive immunization with AMEV2 antisera and in vitro opsonophagocytic killing assays (OPKAs) were used to assess the critical role of antibody-mediated protection induced by AMEV2 vaccination. Results: The passive transfer of AMEV2 immune sera to naïve mice afforded 67% protection in a pulmonary challenge mouse model. Although AMEV2 sera reacts with bacterial antigens, it is not bactericidal on its own and does not enhance the complement-mediated direct killing of *A. baumannii*. However, OPKAs demonstrate AMEV2 sera enhancement of the killing of *A. baumannii* in the presence of primary bone marrow-derived macrophages. This killing occurs via complement and Fc gamma receptor-mediated phagocytosis. A highly immunogenic AMEV2 component peptide, pTonB, elicits pTonB-specific antibodies and protection in vivo. The depletion of pTonB antibodies from AMEV2 immune sera by pTonB absorption significantly reduced the opsonophagocytic killing of *A. baumannii* in vitro. Conclusions: The data presented here demonstrate the importance of humoral immunity and its protective mechanisms against *A. baumannii*. These findings further expand the in vivo evaluation of in silico-designed vaccines as a viable alternative to combat the current global MDR pathogen health crisis.

## 1. Introduction

*Acinetobacter baumannii*, a nosocomial opportunistic Gram-negative coccobacillus bacterium, is a member of the ESKAPE group of pathogens [1]. These pathogens notably exhibit a multidrug-resistant (MDR) phenotype underscoring the pressing need for new and effective interventional agents [2]. Infections with MDR *A. baumannii* result in increased patient mortality and healthcare costs [3]. Its nosocomial nature affects intensive care units globally due to its ability to “persist and resist”, avoiding desiccation on healthcare surfaces. This environmental persistence subsequently leads to ventilator-associated pneumonia, urinary tract infections, catheter-related bacteremia, skin and soft tissue infections, and wound infections in military personnel [4,5,6,7,8,9]. Increased incidence of MDR *A. baumannii* contributes to the ineffective use of first-line antibiotics to treat infections caused by recent clinical isolates [10]. This leaves only last-resort treatment options like carbapenems and colistin as a treatment strategy in the clinic despite their toxicity to the patient [11]. However, the emergence of extreme drug-resistant (XDR) phenotypes to even these last-resort antibiotics has increased in recent *A. baumannii* isolates [12].

The emergence of multidrug-resistant *A. baumannii* prompted the World Health Organization (WHO) to assign carbapenem-resistant *A. baumannii* (CRAB) the highest priority ranking in 2017, necessitating the development of novel drug therapies for control [13]. More recently, WHO published its Bacterial Priority Pathogen List for 2024 with CRAB remaining a critical group pathogen due to a continued high mortality rate, extremely low treatability, and the unlikely outlook for new treatment options in the next 5–7 years [14]. These global calls for alternative solutions to this public health crisis underscore the need to develop effective immunotherapeutics to combat this pathogen.

Prophylactic vaccination could prove effective in managing MDR infections in high-risk susceptible populations. Previously, our laboratory characterized a live attenuated strain of *A. baumannii* deficient in thioredoxin [15]. Vaccination afforded mice 100% protection against a 10× LD_50_ systemic challenge. Passive immunization with vaccinated sera also protected naïve mice, suggesting antibody-mediated protection as the primary protective correlate. Despite robust protection, there is hesitancy to vaccinate using a live bacterium. Thus, our laboratory has focused on developing safer protein subunit vaccines against this pathogen.

Our effort has focused on developing an immunoinformatics-based multi-peptide vaccine capable of eliciting an immune response to multiple *A. baumannii* virulence factors involved in pathogenesis [16]. The *Acinetobacter* Multi-Epitope Vaccine (AMEV2) is comprised of an *A. baumannii* thioredoxin leader protein linked to five peptides (ranging from 42 to 57 amino acids) derived from a copper resistance protein (pNlpE), an outer membrane nuclease (pNucAB), a siderophore receptor (pTonB), a zinc piracy receptor (pZnuD), and an outer membrane protein (pOmp38) [17,18,19,20,21,22]. Immunization with this multi-peptide construct using the AddaS03 adjuvant system resulted in robust humoral and cell-mediated responses [16]. A high frequency of AMEV2-specific antibody-secreting cells was observed in the spleens and bone marrow of immunized mice. Additionally, significant IgG1 antibody titers were elicited to the construct as well as the respective peptide components. Furthermore, screening of the AMEV2 construct with sera from *A. baumannii*-infected mice demonstrated antibody reactivity to its respective epitopes, supporting its relevance to natural infection, i.e., in vivo peptide antigenicity.

Our previous study evaluated the protective efficacy of AMEV2 as a potential vaccine candidate against *A. baumannii* pulmonary infections. AMEV2 vaccination afforded 60% protection to mice against pulmonary infection with a lethal dose of a virulent *A. baumannii* clinical isolate (Ci79). AMEV2-vaccinated mice exhibited significantly reduced bacterial burdens 48 h post-challenge compared to adjuvant-only mice. Additionally, AMEV2 vaccination provided 80% protection against systemic infection induced by intraperitoneal challenge with 4× LD_50_ of *A. baumannii* strain AB5075. Because of the humoral immunity exhibited, we hypothesize that AMEV2 antibodies can protect against *A. baumannii* infections. In this study, we conducted passive immunization to test our hypothesis and further delineated the mechanism of this antibody-mediated protection. The data presented here support the usefulness of immunoinformatics-based vaccines as potential therapeutic agents against the growing threat of current and emerging MDR pathogens.

## 2. Materials and Methods

### 2.1. Animals

Animal experiments were performed using 7–8-week-old mice. C57BL/6 mice were purchased from Jackson Laboratories (Bar Harbor, ME, USA). B6.129P2-*Fcer1g^tm1Rav^*N12 mice were purchased from Taconic Biosciences (Germantown, NY, USA). All animal experiments were performed in accordance with Institutional Animal Care and Use Committee Protocol MU070 at the University of Texas at San Antonio’s AAALAC-accredited animal facility.

### 2.2. Bacteria

*Acinetobacter baumannii* type strain ATCC 19606 was purchased from American Type Culture Collection (ATCC, Manassas, VA, USA). *A. baumannii* clinical isolate 79 (Ci79) obtained from the San Antonio Military Medical Center (SAMMC; Fort Sam Houston, San Antonio, TX, USA) was provided by Dr. James Jorgensen (University of Texas Health Science Center at San Antonio, San Antonio, TX, USA) [23,24].

### 2.3. Bacterial Culture

Frozen glycerol stocks of *A. baumannii* were streak-plated on Luria–Bertani (LB) agar plates supplemented with ampicillin (100 µg/mL) and grown overnight at 37 °C. The following day, a single colony was incubated overnight at 37 °C in LB broth. The bacterial culture was then subcultured the following day to an OD_600 nm_ = 0.03 in fresh LB broth and grown for 3.5 h until the mid-log phase. The cultures were centrifuged at 5000 rpm for 5 min, and bacterial pellets were resuspended and washed in phosphate-buffered saline (PBS). The bacteria were diluted to an OD_600 nm_ = 0.5 (≈2 × 10^8^ CFU/mL). These cultures were either diluted for in vitro assays or further centrifuged and concentrated for intranasal (i.n.) challenge. Bacterial challenge inoculum CFU/mL was determined by serial dilution and plating.

### 2.4. Sera Generation

The same vaccination schedule previously used for active vaccination was followed for serum generation for passive vaccination with C57BL/6 mice (*n* = 5 per group) [16]. The recombinant AMEV2 protein resuspended in PBS (200 µg/mL) was mixed 1 to 1 with the AddaS03 adjuvant (InvivoGen, San Diego, CA, USA) and vortexed thoroughly to create a 100 µg/mL mixture. The mice were subcutaneously injected with either a mock control vaccination of 100 µL of PBS + AddaS03 or 10 µg of rAMEV2 + AddaS03. Vaccinations were administered on days 0, 14, and 28. Vaccinated mice were terminally bled for serum collection two weeks after the second boost. Some blood samples of the fully vaccinated mice were pooled, and the serum was then aliquoted and stored at −80 °C for up to 12 weeks. The serum had one freeze–thaw total before being used in passive vaccination, complement, and opsonophagocytic killing assays.

### 2.5. Passive Sera Vaccination and Pulmonary Challenge

C57BL/6 mice (*n* = 6 per group) were injected intraperitoneally with 100 µL of sera from either adjuvant only or AMEV2-vaccinated mice 24 h before intranasal challenge. The pulmonary challenge was carried out as previously described [16]. The mice were monitored daily for weight loss and morbidity for 14 days and total survival for 30 days.

### 2.6. Complement Killing Assay

Control and AMEV2 sera pools were heat-inactivated at 56 °C for 30 min to remove the complement. Heat-inactivated sera pools were diluted 1 to 10 with PBS, and 50 µL was added to a 96-well plate in quadruplicate. An amount of 25 µL of either freshly reconstituted baby rabbit sera or heat-inactivated baby rabbit sera was added to corresponding wells. Then, 15 µL of PBS was added to all wells. Lastly, Ci79 or ATCC 19606 strains of *A. baumannii* were grown and diluted to approximately 5 × 10^4^ CFUs with PBS. In total, 10 µL (~500 CFUs) of each strain was added to the wells to make the final reaction volume 100 µL. The prepared plate was incubated at 37 °C for 1 h and 30 min. The reaction was quenched with 100 µL of ice-cold PBS added to all wells. The wells were then serially diluted and plated to enumerate viable bacteria.

### 2.7. Determination of Antibody Levels by ELISA

ELISAs were carried out as previously described [16]. The only difference was in coating the plates with either 10^6^ UV-inactivated *A. baumannii* or AMEV2 individual peptides (Peptide 2.0, Chantilly, VA, USA and GenScript, Piscataway, NJ, USA). Endpoint titers were determined as the highest dilution with an absorbance reading of 0.1 greater than the blank absorbance reading.

### 2.8. Bone Marrow-Derived Macrophage Generation

C57BL/6 or B6.129P2-*Fcer1g^tm1Rav^*N12 mice were sacrificed, and their two hind limbs were collected for bone marrow isolation. In total, 5 × 10^6^ bone marrow cells were seeded into 100 mm × 15 mm Petri dishes in 10 mL of complete R10 media (RPMI 1640, 10% fetal bovine sera, 1% Pen–Strep, 1% L-glutamine) supplemented with 20 ng/mL of macrophage colony-stimulating factor (M-CSF) (Gibco/Thermo Fisher Scientific, Waltham, MA, USA). On day 3, an additional 10 mL of R10 supplemented with 20 ng/mL of M-CSF was added to the Petri dish. On day 6, 10 mL of media was removed from the Petri dish, and 10 mL of R10 supplemented with 20 ng/mL of M-CSF was added. On day 8, the cells were fed one last time, with the removal of 10 mL and then the addition of 10 mL of supplemented media. On day 9, the media were removed from the Petri dishes, and PBS was added to wash away the nonadherent and semi-adherent cells. The remaining adhered cells were collected with Accutase cell detachment solution (StemCell, Vancouver, BC, Canada). The Accutase reaction was quenched and cells were collected, spun down, and counted for plating. The macrophages were seeded into the experimental plates and incubated with 20 ng/mL of M-CSF overnight to allow recovery from the Accutase treatment before being used in downstream in vitro applications.

### 2.9. Opsonophagocytic Killing and Bacterial Uptake Assays

A reaction volume of 100 µL containing 50% diluted *A. baumannii*, 37.5% freshly reconstituted baby rabbit sera, and 12.5% immunized sera was incubated at 37 °C for 30 min for opsonization. The opsonized bacteria were then diluted with R10 without antibiotics to 10^5^ CFU/mL. Wells containing 10^5^ adhered BMDMs were washed with R10 without antibiotics, and 100 µL of the opsonized bacteria was pipetted into the wells with or without the macrophages. Plates were spun down at 300× *g* for 5 min and then placed in the 37 °C, 5% CO_2_ incubator for 1 h. The plate was gently tapped on all sides to mix every 15 min. Following the incubation, the plate was placed on an ice pack at 4 °C for 20 min to cease macrophage function and bacterial growth. The media in the wells were then serially diluted and plated on LB agar to enumerate the remaining viable bacteria. R10 supplemented with 25 µg/mL of Polymyxin B (MilliporeSigma, St. Louis, MO, USA) was added to all macrophage wells and incubated at 4 °C for 1 h to kill the remaining extracellular bacteria. Following the hour, the cells were washed twice with media and 200 µL of 0.2% Deoxycholate was added to each well to lyse the macrophages. Complete lysis was observed under the tissue culture microscope, and 100 µL was removed from each well, serially diluted, and plated on LB agar to enumerate the bacteria taken up by the macrophages.

### 2.10. T-Cell ELISpot Assays

T-cell reactivity to AMEV2 peptides was evaluated using IFNγ, IL-4, and IL-5 ELISpot assays as previously described with minor adjustments [16]. Splenocytes were evaluated for in vitro recall with each AMEV2 peptide (2 µM). pNspec (2 µM), a *Coccidioides posadassii* peptide, served as a specificity control. PVDF membrane ELISpot plates (MilliporeSigma, St. Louis, MO, USA) were coated overnight with IFNγ (clone: AN-18, 2 µg/mL), IL-4 (clone: 11B11, 4 µg/mL), or IL-5 (clone: TRFK5, 5 µg/mL) capture antibodies. Biotinylated IFNγ (clone: R4-6A2, 0.5 µg/mL), IL-4 (clone: BVD6-24G2, 2 µg/mL), or IL-5 (clone: TRFK4, 0.5 µg/mL) were used as detection antibodies.

### 2.11. AMEV2 Peptide-Specific Antibody Absorption

Immuno-Blot PVDF membranes (Bio-Rad, Hercules, CA, USA) were pre-wet with methanol and washed with water. An amount of 100 µL (590 µg) of either the pNspec or pTonB peptide was pipetted onto separately activated membranes. The membranes were incubated at 4 °C overnight. The following day, the membranes were washed with PBS and then transferred to tubes containing 1% bovine sera albumin (BSA) for 1 h at room temperature to block. After blocking, the membranes were each washed once more with PBS before being transferred to tubes containing AMEV2 sera. The membranes were incubated with the sera for 2 h at room temperature. After 2 h, the membrane was removed from each tube, and the sera were aliquoted and frozen for later in vitro assays. Successful absorption and reduction in peptide antibody titer were confirmed by peptide ELISA.

### 2.12. Statistical Analysis

GraphPad Prism 10.0 was used to determine statistical significance tests. The Shapiro–Wilk normality test was first performed to determine the normal (Gaussian) distribution of samples within a group as the basis of subsequent pairwise comparison using either parametric or nonparametric tests as indicated in the figure legend for each experiment. Differences between adjuvant mock control and AMEV2 vaccinated groups, as well as control and AMEV2 sera-treated bacteria, were assessed using the Student’s *t*-test, One-way ANOVA, and Two-way ANOVA. Survival rates were analyzed with the Log-rank Mantel–Cox test. For multi-group comparisons using ANOVA, both the *p*-value for the overall ANOVA and the *p*-values for the pairwise comparisons were reported. Differences were considered statistically significant when *p* ≤ 0.05 and the actual *p*-value was reported.

## 3. Results

### 3.1. AMEV2 Immune Sera Protect Mice Against A. baumannii Infections

**Figure 1:**
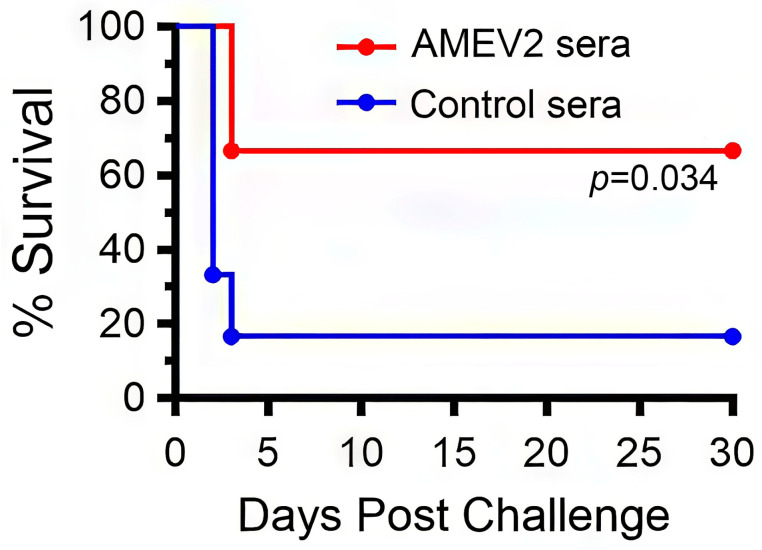
AMEV2 immune sera provides passive protection against pulmonary *Acinetobacter baumannii* infection. C57BL/6 (7–8-week-old) mice (*n* = 6 per group) were injected intraperitoneally with 100 µL of either control or AMEV2 sera. At 24 h post-injection, mice were challenged intranasally with 10^8^ CFUs of Ci79 *A. baumannii* and monitored for 30 days for survival. Log-rank Mantel–Cox test between control and AMEV2 passively vaccinated mouse survival status over 30 days.

We have previously demonstrated that active AMEV2 vaccination protected mice against pulmonary as well as systemic *A. baumannii* infection [16]. To further investigate the protective immunity associated with AMEV2 protection, we determined if antibodies alone could protect against *A. baumannii* infection in vivo. Sera were obtained from either AMEV2-vaccinated C57BL/6 mice (AMEV2 sera) or adjuvant-only treated mice (control sera). Naïve C57BL/6 mice (*n* = 6 per group) were injected intraperitoneally with either 100 µL control or AMEV2 sera. Mice were anesthetized 24 h post-injection and challenged intranasally with 50 µL of PBS containing 10^8^ CFUs of Ci79 (Figure 1). Four of six mice passively immunized with AMEV2 sera survived the pulmonary challenge versus only one of the control sera-injected mice. Thus, the passive vaccination of naïve mice with AMEV2 sera provides antibody-mediated protection against *A. baumannii* infection in vivo.

### 3.2. AMEV2 Immune Sera Enhanced Opsonophagocytic Killing of A. baumannii

**Figure 2:**
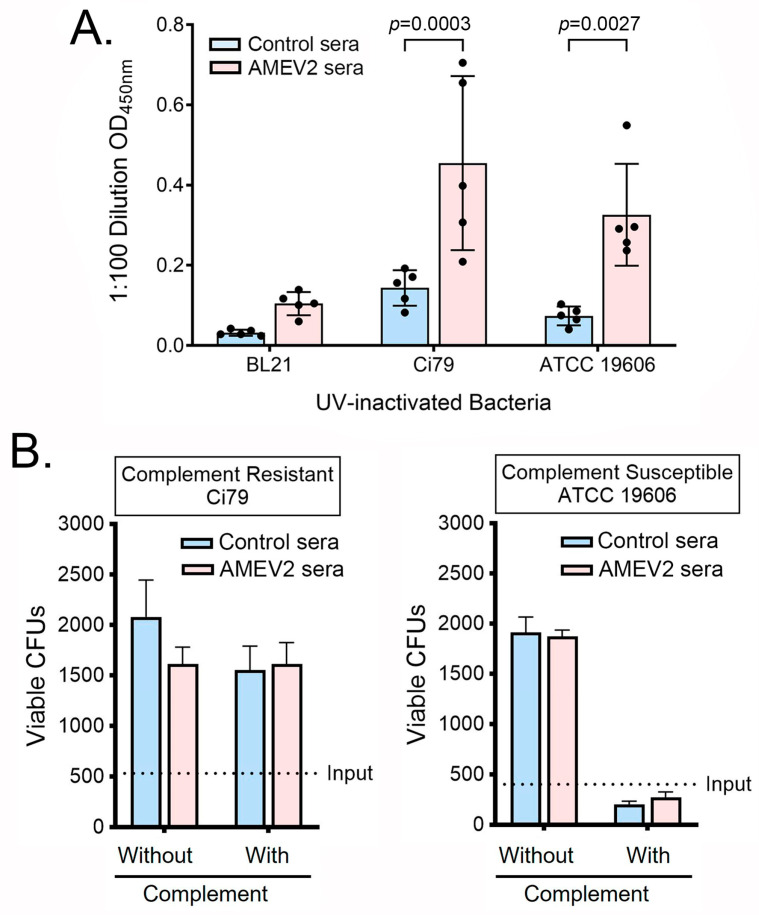
AMEV2 vaccination generates antibodies to *A. baumannii* but does not enhance complement-mediated killing. (**A**) Control and AMEV2 sera (*n* = 5 per group) were diluted 1:100 and checked for reactivity with whole cell UV-inactivated BL21 *E. coli* and two *A. baumannii* strains by indirect ELISA. Data are presented as the mean ± SD. Two-way ANOVA (*p* = 0.0471) with Sidak’s multiple comparisons test. (**B**) Ci79 or ATCC 19606 *A. baumannii* was incubated with heat-inactivated control sera or heat-inactivated AMEV2 sera in the presence of baby rabbit sera containing either intact or heat-inactivated complement in quadruplicate. Following incubation (1.5 h) at 37 °C, wells were serially diluted and plated to enumerate viable bacteria.

We further delineated the protective mechanisms of AMEV2 sera. The in vitro reactivity of control and AMEV2 sera was screened against two strains of *A. baumannii* by indirect ELISA (Figure 2A). AMEV2 sera exhibited significant reactivity to the clinical isolate (Ci79) and ATCC type strain (19606) while control sera did not. *E. coli* (BL21), a Gram-negative bacterium used in the expression and purification of AMEV2, was included as a nonspecific control for specificity. The results showed minimal reactivity of *E. coli* with both control and AMEV2 sera. These results indicated the presence of anti-*Acinetobacter*-specific antibodies in the AMEV2 sera. We then evaluated the ability of the sera to inhibit bacterial growth via complement mediation, as it is known that the early reactants (C1, C4, C2) in the classical complement pathway in mice are extremely unstable [25]. Thus, an exogenous baby rabbit serum was used as the complement source in complement assays for killing, growth, and opsonophagocytosis. *A. baumannii* Ci79 (a complement-resistant strain) and ATCC 19606 (a complement-susceptible strain) were incubated with pooled control or AMEV2 sera in the presence of either intact or heat-inactivated baby rabbit complement. As shown in Figure 2B, both Ci79 and 19606 strains replicated in the sera without functional complement. The complement-resistant *A. baumannii* Ci79 strain exhibited a slight but not statistically significant reduction in bacterial growth when incubated with AMEV2 sera compared to treatment with control sera. In the presence of intact complement, incubation with AMEV2 sera did not significantly increase Ci79 killing when compared to control sera incubation. In contrast, the growth of the complement-sensitive ATCC 19606 strain was markedly reduced to a similar extent by both control and AMEV2 sera in the presence of intact complement. Collectively, in the presence of complement, AMEV2 sera did not abrogate complement resistance in the Ci79 strain nor enhance complement killing in the complement-susceptible ATCC 19606 strain. Thus, the protective mechanism of the AMEV2 sera is not associated with classical complement-activation-mediated bactericidal events.

**Figure 3:**
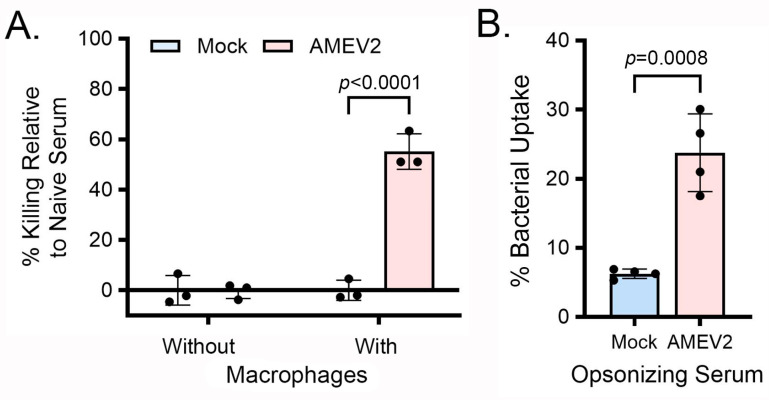
AMEV2 immune sera enhance the opsonophagocytic killing of *A. baumannii* and increase bone marrow-derived macrophage bacterial uptake. (**A**) Ci79 bacteria were opsonized in the presence of heat-inactivated naïve mouse sera or pooled control or AMEV2 sera and fresh baby rabbit sera as a complement source for 30 min at 37 °C. Then, 10^4^ CFUs of opsonized bacteria were seeded into wells with or without 10^5^ adhered BMDMs. Following a 1 h incubation, the remaining viable bacteria were diluted and plated on LB agar for enumeration. The reduction in bacterial counts by the control and AMEV2 sera treatments was compared to naïve serum treatment and presented as % relative killing. Two-way ANOVA (*p* < 0.0001) with Sidak’s multiple comparisons test. (**B**) Phagocytosed bacteria were released from macrophages by lysing and enumerated by serial dilution and plating on LB agar. Student’s *t*-test. Both results are expressed as the mean ± SD and each is representative of two independent experiments with technical replicates.

Since AMEV2 sera did not enhance direct complement killing, we investigated whether the sera could opsonize the pathogen and subsequently enhance phagocytic uptake using primary bone marrow-derived macrophages (BMDM). An opsonophagocytic killing assay (OPKA) was employed to determine if in vitro antibody reactivity to *Acinetobacter* with AMEV2 sera resulted in opsonization and enhanced killing when incubated with the macrophages (Figure 3A). Bacteria that had been opsonized with AMEV2 sera exhibited approximately 50% reduction in viable Ci79 compared to naïve sera. In contrast, control sera-opsonized bacteria exhibited no difference in killing. When both types of opsonized bacteria were incubated in wells without macrophages, no increase in killing was observed, indicating no sera bactericidal activity. Following incubation, macrophages were lysed, and bacterial uptake was enumerated (Figure 3B). Macrophages incubated with AMEV2 sera-opsonized bacteria exhibited a significant increase in bacterial uptake compared to that of control sera. Thus, AMEV2 sera enhance the opsonophagocytic killing of *A. baumannii* by the enhancement of primary bone marrow-derived macrophage recognition and the uptake of the pathogen in vitro.

**Figure 4:**
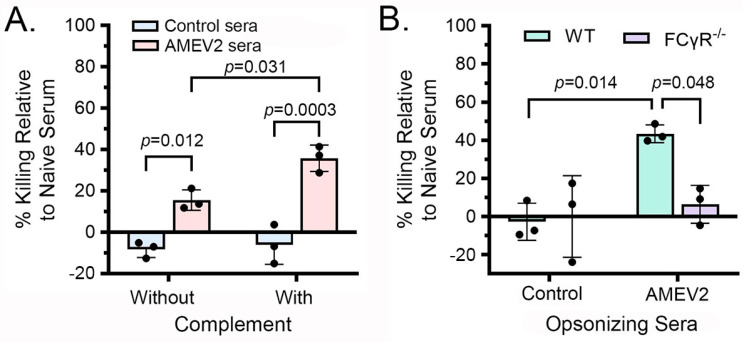
AMEV2 sera opsonophagocytic killing is mediated by enhancing classical complement activation and FCγ receptor-mediated phagocytosis. (**A**) Ci79 bacteria were opsonized in the presence of heat-inactivated naïve mouse sera or pooled control or AMEV2 sera with or without complement. Opsonized bacteria (10^4^ CFU) were seeded into wells containing 10^5^ BMDM. Following a 1 h incubation, the remaining viable bacteria were diluted and plated on LB agar for enumeration. The reduction in bacterial counts by the control and AMEV2 sera treatments was compared to naïve serum treatment and presented as % relative killing. Results are expressed as the mean ± SD and are representative of two independent experiments with technical replicates. Two-way ANOVA (*p* = 0.0429) with Sidak’s multiple comparisons test. (**B**) Ci79 (10^4^ CFU) bacteria opsonized in the presence of heat-inactivated naïve mouse sera or pooled control or AMEV2 sera without complement were seeded into wells containing either 10^5^ WT BMDM or FcγR^−/−^ BMDM. Following a 1 h incubation, the remaining viable bacteria were diluted and plated on LB agar for enumeration. The reduction in bacterial counts by the control and AMEV2 sera treatments in WT or FCγR^−/−^ BMDM was compared to naïve serum treatment and presented as % relative killing. The results are expressed as the mean ± SD. Two-way ANOVA (*p* = 0.029) with Sidak’s multiple comparisons test.

The enhancement of opsonophagocytic killing by AMEV2 sera appears to be the primary mechanism of antibody-mediated protection in AMEV2-vaccinated mice. Therefore, we investigated whether this killing was facilitated by the activation of the classical complement pathway or primarily by Fc receptor-mediated uptake. An identical OPKA experiment was performed using bacteria opsonized in the presence of either an intact complement source or a heat-inactivated complement source (Figure 4A). A significantly higher amount of AMEV2 sera-opsonized Ci79 was killed compared to control sera-opsonized bacteria when incubated with bone marrow-derived macrophages even in the absence of complement. The addition of intact complement further enhanced the killing of AMEV2 but not control sera-opsonized Ci79. These results, along with the lack of complemented-mediated direct bacterial killing previously observed, support the AMEV2 antibody activation of the classic complement pathway enhancing macrophage pathogen recognition. The difference in bacterial killing between AMEV2- and control sera-opsonized Ci79 in the absence of an intact complement implies complement-independent opsonophagocytic killing mechanisms. Thus, we investigated the role of Fc receptors in AMEV2 sera-mediated opsonophagocytic killing. The OPKAs were carried out on wells containing either WT BMDMs or BMDMs from B6.129P2-*Fcer1g^tm1Rav^*N12 mice, which are deficient in the gamma chain subunit of the FcγR1, FcγRIII, and FceR1 receptors (Figure 4B). In this experiment, the complement source and vaccinated sera were heat-inactivated to negate complement-mediated phagocytosis. WT BMDMs continued to demonstrate the significant killing of bacteria opsonized with AMEV2 sera, whereas killing was significantly reduced and/or absent when the AMEV2 sera-opsonized bacteria were incubated with BMDMs deficient in FCγRs. These data suggest that AMEV2 antibodies opsonize the bacteria, thus enhancing killing through complement- and Fcγ receptor-mediated phagocytosis.

### 3.3. Anti-pTonB Antibody Enhanced Opsonophagocytic Killing of A. baumannii

**Figure 5:**
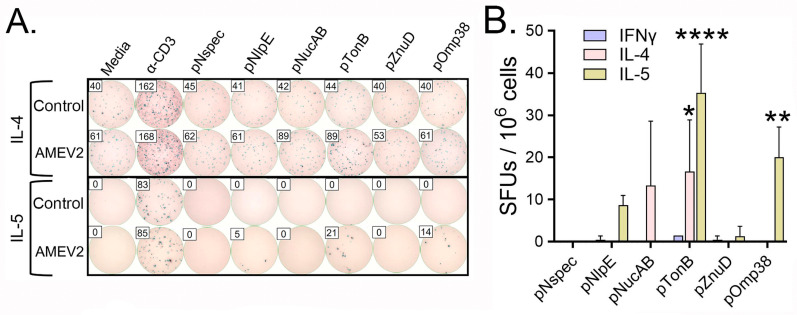
AMEV2 vaccination induces Th2 cellular immune responses. Seven- to eight-week-old C57BL/6 mice (*n* = 3 per group) were vaccinated subcutaneously with either PBS + AddaS03 (mock control) or rAMEV2 (10 µg) +AddaS03 (AMEV2) on days 0, 14, and 28, and rested for 4 weeks prior to spleen removal. (**A**) Representative ELISpot readouts (each well was seeded with 5 × 10^5^ splenocytes) for control or AMEV2-vaccinated splenocytes stimulated with media, α-CD3, nonspecific peptide (pNspec), and rAMEV2 component peptides. (**B**) Detection of IFNγ, IL-4, and IL-5 spot-forming units (SFU) to AMEV2 peptides. Data are presented as the mean *±* SD. * *p* = 0.0114, ** *p* = 0.0015, **** *p* ≤ 0.0001. Two-way ANOVA (*p* < 0.0001) with Tukey’s multiple comparisons test between SFUs of indicated AMEV2 peptide and pNspec.

The AMEV2 multi-peptide construct allows the investigation of which of the five component peptides are necessary for antibody-mediated protection. Previously, we demonstrated that vaccination with AMEV2 resulted in increased IL-4-secreting splenocytes upon restimulation with the AMEV2 peptide construct. The same T-cell ELISpot recall assay was used to determine peptide-specific reactivity when AMEV2-vaccinated splenocytes were restimulated with the respective peptides (Figure 5). AMEV2-vaccinated splenocytes demonstrate significant levels of IL-4 and IL-5 but minimal IFNγ secretion when restimulated with AMEV2 constituent peptides. Splenocytes restimulated with pNlpE, pTonB, and pOmp38 peptides showed increased IL-5 secretion, while pNucAB and pTonB peptide recall exhibited IL-4 secretion. Additionally, no recall of the pNspec peptide was observed. pNspec is a peptide of similar length to AMEV2 peptides but used in fungal *Coccidioides posadassii* studies in our laboratory and was included as a specificity control. Mock (adjuvant only)-vaccinated mice exhibited no recall response to any of the peptides. Of all the peptides tested, pTonB showed the highest frequency of Th2 T cell reactivity.

**Figure 6:**
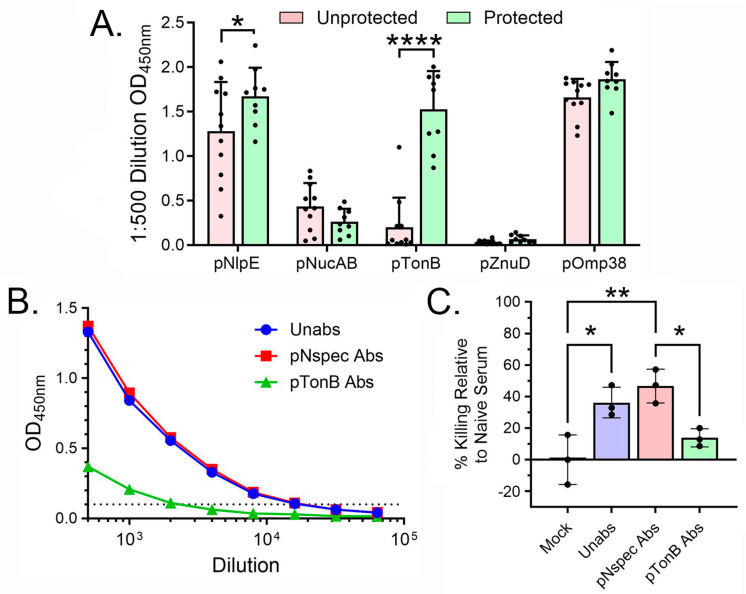
pTonB-specific antibodies appear to be protective in *Acinetobacter baumannii* infections. (**A**) AMEV2 immune sera collected before *A. baumannii* challenge were screened for peptide reactivity with peptide ELISA. The antibody compositions of AMEV2-vaccinated mice that survived the challenge were compared to those that succumbed to infection. * *p* = 0.024, **** *p* < 0.0001. Two-way ANOVA (*p* < 0.0001) with Sidak’s multiple comparisons test. (**B**) Pooled AMEV2 sera were absorbed with either pNspec or pTonB peptides and their pTonB-specific antibody titer was determined by peptide ELISA. The dotted line indicates the endpoint titer determination threshold of an OD_450 nm_ value greater than 0.1. (**C**) The unabsorbed and absorbed AMEV2 vaccinated sera killing were compared by the opsonophagocytic killing assay. The reduction in bacterial counts by AMEV2 sera treatments was compared to naïve serum treatment and presented as % relative killing. Results are expressed as the mean ± SD and are representative of two independent experiments with technical replicates. * *p* ≤ 0.05, ** *p* = 0.0038. One-way ANOVA (*p* = 0.003) with Tukey’s multiple comparisons test.

The variability of peptide-specific antibody generation in each mouse prompted our investigation of the in vivo importance of each peptide across two protection studies (*n* = 20 mice) (Figure 6A). AMEV2-vaccinated mice that were protected from *A. baumannii* challenge had significantly more pTonB-specific antibodies compared to those that were unprotected. pNlpE-specific antibody also showed an increase in protected mice, but it was not as striking as that observed for the pTonB antibody. To evaluate the importance of pTonB-specific antibodies in the OPKA, we absorbed AMEV2 sera with pTonB to reduce the pTonB-specific antibody titer eightfold from 1:16,000 to 1:2000 (Figure 6B). The pTonB-absorbed sera in the OPKA showed significantly (*p* = 0.027) reduced killing compared to nonspecifically absorbed sera (Figure 6C). Furthermore, the depletion of the pTonB antibody resulted in poor opsonophagocytic killing that was not significantly different from unimmunized control sera. These combined in vivo and in vitro data indicate that the pTonB peptide contains epitopes important for antibody-mediated protection against *A. baumannii*.

## 4. Discussion

Given the urgent need to develop new antimicrobials to combat the rise of MDR pathogens, researchers have begun to focus on immunotherapeutics as a viable solution to this global health problem. Previously, we demonstrated the in vivo effectiveness of an immunoinformatics-based multi-peptide vaccine (AMEV2) for protection against *A. baumannii* infection [16]. Reverse vaccinology has also been applied to develop *Acinetobacter* multi-peptide vaccines by others [26,27]. Vaccination with a rOmp22 vaccine, which consists of three B-cell and two T-cell epitopes encapsulated in chitosan-PLGA nanoparticles, provided robust protection (60–70%) against pulmonary MDR *A. baumannii* infection [26]. A multi-epitope assembly peptide (MEP) vaccine containing B- and T-cell epitopes from FilF (pilus assembly protein), NucAb (outer membrane nuclease), and Ata (trimeric autotransporter protein) also provided potent protection (90%) against the *A. baumannii* type strain in a mouse model of intraperitoneal challenge [27]. The multicomponent vaccines, such as AMEV2 and MEP, can target different virulence factors of *A. baumannii* to enhance the vaccine’s efficacy.

Due to the acute nature of the experimental challenge model of *A. baumannii* and previous vaccine research germane to this pathogen, it was hypothesized that the protective mechanism of AMEV2 was primarily antibody-mediated. The robust humoral immune response demonstrated in our previous study was tested for reactivity against UV-inactivated whole *A. baumannii* bacteria using AMEV2 sera. Here, we showed that vaccination successfully generates antibodies that recognize antigens on both the clinical isolate strain used in our protection studies as well as the ATCC type strain.

In vitro assays were utilized to delineate whether AMEV2 antibodies function to kill or opsonize *A. baumannii*. The complement system bridges the innate and adaptive immune systems in defense against extracellular pathogens. Variation in complement susceptibility has been well characterized in *A. baumannii* isolates throughout the years [28]. Early isolates showed susceptibility while most recent clinical isolates are complement-resistant likely due to their capsule [29]. When AMEV2 sera were incubated with intact complement, it did not inhibit Ci79 complement resistance nor enhance the killing of the complement-susceptible ATCC 19606 strain. Additionally, no difference in bacterial growth in the absence of complement was observed, indicating no inhibition of bacterial growth in the presence of these antibodies. Additionally, AMEV2-elicited antibodies do not enhance the formation of the membrane attack complex (MAC) and the subsequent lysis of *A. baumannii*. The lack of bactericidal activity of vaccinated sera against *A. baumannii* is consistent with other studies [30].

Despite exhibiting no bactericidal activity against the pathogen, AMEV2 antisera enhances the killing of *A. baumannii* when incubated with primary bone marrow-derived macrophages (BMDM) in vitro. This killing is diminished, albeit still significant, when the complement is removed from the assay, indicating that despite the pathogen’s inhibition of MAC formation, AMEV2 antibodies recognize the pathogen and activate the classical complement pathway, leading to complement-mediated phagocytosis. The reduction in opsonophagocytic killing in the absence of complement has been observed in other *A. baumannii* vaccines [31,32,33]. The use of primary BMDMs generated from mice deficient in FC gamma receptors revealed the killing of the bacterium to be facilitated through the recognition of the FC region of bound AMEV2 antibodies by the FCγR of BMDM. In the absence of complement, WT BMDM still demonstrated the significant killing of bacteria when opsonized with AMEV2 antisera. However, killing was not observed when incubated with FCγR^−/−^ BMDM. Thus, we are one of the first to demonstrate the specificity of opsonophagocytic killing in vitro. However, the whole-cell intranasal vaccination study by KuoLee et al. used FCγR^−/−^ mice in vivo to elucidate its protective mechanism [34]. To their surprise, FCγR^−/−^-vaccinated mice were protected in an intranasal challenge, indicating that FC gamma receptor-mediated opsonophagocytosis is unnecessary in the mucosal vaccination regimen. However, complement-mediated opsonophagocytosis alone may be sufficient for antibody-mediated protection against *A. baumannii* following parenteral vaccination, as in this study.

The success of passive immunization and monoclonal antibodies against *A. baumannii* has demonstrated antibody-mediated protection to be of paramount importance in controlling infections of this extracellular pathogen [35,36,37,38,39]. Congruent with this thinking, we passively transferred control and AMEV2 sera to naïve mice before intranasal challenge with *A. baumannii*. Mice given AMEV2 sera 24 h prior to the challenge received partial protection with 67% survival compared to the passively immunized mock mice with only 17% survival. Previously, one vaccine was shown not to afford protection via passive immunization [34]. KuoLee et al. showed that intranasal vaccination with formalin-killed *A. baumannii* protected against intranasal challenge; however, when vaccination-derived serum was passively transferred to naïve mice, they were unprotected. This suggests that a parenteral vaccination likely generates a better antibody response for passive transfer than one that solely stimulates mucosal immunity.

Of the five peptides contained in AMEV2, the data presented here indicate that antibodies generated against the pTonB peptide significantly enhance the opsonophagocytic killing of *A. baumannii* in vitro. When evaluating AMEV2 peptide-specific immunogenicity by ELISpot, we observed significant IL-4 and IL-5 recall responses in AMEV2 splenocytes reexposed to the pTonB peptide. This strong Th2 response results in pTonB-specific IgG1 antibodies which are ideal for the identification of an extracellular pathogen through enhanced binding to C1q and Fcγ receptors [40]. Along with the high immunogenicity of this peptide, our in vivo data also demonstrate a correlation between protection and the amount of pTonB-specific antibody generated in AMEV2 vaccinated mice. The acute nature of this infection indicates how successful this pathogen is at growing within the host, in which a significant part involves siderophore receptors and iron acquisition [41]. Therefore, antibodies targeting this essential function result in protection in an acute model. In addition to pTonB, the protective AMEV2 serum also contains high levels of anti-pNlpE and anti-omp38 antibodies. The *Acinetobacter* NlpE (GenBank WP_000749178.1) is an uncharacterized lipoprotein that shares homology with the *E. coli* copper resistance CutF protein [42]. The outer membrane protein Omp38 is capable of inducing apoptosis in epithelial cells [21], and Omp38-specific mAb treatment can reduce *A. baumannii* infection in mice [43]. The inclusion of pTonB, pNlpE, and pOmp38 in the next generation of AMEV vaccine design could provide robust protective antisera against MDR *Acinetobacter*.

## 5. Conclusions

In this report, we provided experimental evidence and demonstrated the protective role of AMEV2 immune sera against *A. baumannii*, in part by enhancing the opsonophagocytic killing of the bacteria. Most currently approved vaccines against infectious diseases contain either inactivated or live attenuated whole pathogens that stimulate a robust immune response. Subunit vaccines consisting of proteins or peptides are considered a safer choice for the general population, including those who are pregnant, but with some limitations. These subunit vaccines, including AMEV2 (in a multi-peptide format), tend to be less immunogenic and require proper adjuvants and multiple vaccinations to achieve the desired protective efficacy. Furthermore, the peptide vaccines usually require conjugation to a carrier protein and might suffer from confirmational-dependent epitope issues resulting in subpar antibody reactivity. The results of this reported study provide insights into further improvement in AMEV-based vaccine design. First, we confirm that epitope-specific antibodies are critical for protection against *A. baumannii*. Thus, the formulation of AMEVs with Th2-biased adjuvants that stimulate robust Th2 cell activation and antibody production should further enhance vaccine efficacy. The potential adjuvant candidates can be searched in the Vaccine Adjuvant Compendium (VAC) website established by The National Institute of Allergy and Infectious Diseases (NIAID). Second, we have confirmed the well-known observation that not all epitopes in subunit vaccines are equally immunogenic. The immunodominance issue is particularly important when developing peptide vaccines that focus on a limited number of critical epitopes. Thus, the identification of the protective pTonB epitope allows the further refining of AMEV vaccine design. A two-pronged approach will be taken to further explore pTonB-based immunotherapeutics for clinical application. First, for prevention, we will develop a pTonB-dominant vaccine against *Acinetobacter* infection. In general, short peptide vaccination does not induce robust immunity for desired protection, thus, we will construct an AMEV7 vaccine consisting of a TrxA leader protein followed by three copies of pTonB. Second for therapy, with the protective pTonB epitope determined, we can now begin the pursuit of generating a monoclonal antibody to the peptide, affording an additional immunotherapeutic option for the treatment of *A. baumannii* infections.

## Data Availability

Data are contained within the article.
